# Using the Health Belief Model to Explain Mothers’ and Fathers’ Intention to Participate in Universal Parenting Programs

**DOI:** 10.1007/s11121-016-0696-6

**Published:** 2016-09-17

**Authors:** Raziye Salari, Ania Filus

**Affiliations:** 1 Child Health and Parenting (CHAP), Department of Women’s and Children’s Health, Uppsala University, Akademiska sjukhuset (Munin) CHAP, Uppsala, 751 85 Sweden; 2 Center for Self-Report Science, University of Southern California, Los Angeles, USA; 3 Department of Psychology, The University of Queensland, Brisbane, Australia

**Keywords:** Universal parenting programs, Intention to participate, Health Belief Model, Fathers, Mothers

## Abstract

Using the Health Belief Model (HBM) as a theoretical framework, we studied factors related to parental intention to participate in parenting programs and examined the moderating effects of parent gender on these factors. Participants were a community sample of 290 mothers and 290 fathers of 5- to 10-year-old children. Parents completed a set of questionnaires assessing child emotional and behavioral difficulties and the HBM constructs concerning perceived program benefits and barriers, perceived child problem susceptibility and severity, and perceived self-efficacy. The hypothesized model was evaluated using structural equation modeling. The results showed that, for both mothers and fathers, perceived program benefits were associated with higher intention to participate in parenting programs. In addition, higher intention to participate was associated with lower perceived barriers only in the sample of mothers and with higher perceived self-efficacy only in the sample of fathers. No significant relations were found between intention to participate and perceived child problem susceptibility and severity. Mediation analyses indicated that, for both mothers and fathers, child emotional and behavioral problems had an indirect effect on parents’ intention to participate by increasing the level of perceived benefits of the program. As a whole, the proposed model explained about 45 % of the variance in parental intention to participate. The current study suggests that mothers and fathers may be motivated by different factors when making their decision to participate in a parenting program. This finding can inform future parent engagement strategies intended to increase both mothers’ and fathers’ participation rates in parenting programs.

**Electronic supplementary material:**

The online version of this article (doi:10.1007/s11121-016-0696-6) contains supplementary material, which is available to authorized users.

## Introduction

Parent training programs based on social learning models have been evaluated rigorously in numerous studies and have been shown to be effective in promoting positive changes in both parent and child behaviors (e.g., Sanders et al. 2014). More specifically, they are identified as one of the best evidence-based treatments for children and youth with externalizing problems such as disruptive behaviors and ADHD (Evans et al. 2014; Sanders et al. 2014). However, low rates of parental attendance, especially among fathers (Panter-Brick et al. 2014), limit the utility of these programs, particularly when they are offered universally as preventative interventions.

Despite the importance of increasing attendance rates, relatively little research has been conducted on examining factors related to parental motivation to enroll and engage in parenting interventions. First, there is a paucity of research guided by a theoretical framework, as the majority of existing studies have focused on variables of convenience (Morawska and Sanders 2006). Second, little is known about how the gender of the parent affects their motivation to enroll and engage in parenting programs. The majority of existing studies have either solely focused on mothers or aggregated mothers’ and fathers’ data (e.g., Eisner and Meidert 2011). To inform the development of more effective recruitment strategies, the present study investigated factors related to mothers’ and fathers’ willingness to participate in parenting programs using the Health Belief Model as a theoretical framework.

### The Health Belief Model and Parental Intention to Participate in Parenting Programs

The Health Belief Model (HBM; Rosenstock et al. 1988) is one of the most widely used conceptual frameworks for explaining, predicting, and influencing health-related behavior and has received empirical support from both prospective and retrospective studies (see Janz et al. 2002). The HBM has also been directly used to explain factors related to parents’ intention to enroll in parenting programs, as well as their actual participation (Spoth and Redmond 1995; Spoth et al. 1997; Spoth et al. 2000; Thornton and Calam 2011). According to the HBM, parents are most likely to participate in parenting programs if they feel that their children are vulnerable to developing problem behaviors in the future (perceived susceptibility), believe that those problems will have a highly undesirable impact (perceived severity), perceive that parenting programs will be effective in reducing the risk of behavioral problems in their children (perceived benefits), do not find the programs too demanding (perceived barriers), and feel confident that they will be able to utilize what they learn in these programs (perceived self-efficacy). Other factors such as demographic and socio-psychological variables may also affect parents’ participation in parenting programs indirectly by influencing their perception of susceptibility, severity, benefits, and barriers.

Findings from previous studies have generally supported the expected effects of perceived benefits and perceived barriers. These studies have shown that parents who rate parenting programs as more beneficial are more likely to express interest in attending such programs (e.g.,Spoth and Redmond 1995; Thornton and Calam 2011). They have also shown that experience of barriers to participation, such as time constraints and lack of childcare, is negatively related to both inclination to enroll in parenting programs and actual enrollment (e.g., Eisner and Meidert 2011; Spoth and Redmond 1995; Spoth et al. 2000).

The results on perceived susceptibility and severity have been mixed. For example, while in one study (Spoth et al. 1997) parents were more likely to attend a parenting program when they perceived their children to be more susceptible to developing problem behavior in the future, in two other studies, perceived susceptibility was not related to intention to participate (Thornton and Calam 2011) or to actual participation (Bauman et al. 2001). Considering the very small effect size (logistic regression coefficient *B* = 0.02) found in the first study (Spoth et al. 1997), perceived susceptibility may not be a predominant factor in parents’ decisions to participate in parenting programs. This is also true for perceived severity (Bauman et al. 2001; Spoth and Redmond 1995; Thornton and Calam 2011).

In studies of parents’ participation in parenting programs, perceived self-efficacy has often been excluded entirely (e.g., Spoth et al. 2000; Thornton and Calam 2011) or conceptualized as perceived parenting self-efficacy (e.g., Garvey et al. 2006) rather than perceived self-efficacy to carry out the health-related behavior (in the case of parenting programs, to implement the knowledge and strategies taught). This is the case despite the fact that self-efficacy has been hypothesized to be a strong predictor of many health-related behaviors, particularly when the target behavior is more difficult to perform, such as making changes in one’s diet or parenting practices (Rosenstock et al. 1988).

In the HBM research and other research, level of child emotional and behavioral difficulties is one of the socio-psychological factors that have frequently been examined in relation to participation in parenting programs. The results have been mixed, with some studies showing that level of child problems is positively related to intention to attend (e.g., Thornton and Calam 2011) and some studies failing to show such a relation (e.g., Eisner and Meidert 2011). It has been suggested that child problem behaviors may impact parents’ participation in prevention and intervention programs indirectly rather than directly (Spoth and Redmond 1995). Parents who report high levels of emotional and behavioral difficulties in their children may perceive their children to be more vulnerable to experiencing problems in the future and also perceive more benefits associated with attending parenting programs. Therefore, it would seem necessary to investigate both the direct and the indirect effects of perceived child problem behaviors on parental intention to participate in parenting programs.

### Mothers and Fathers

Another factor that has not been previously studied is parent gender. None of the studies mentioned earlier has examined the moderating effect of parent gender, even when the sample included a relatively large percentage of fathers (Spoth and Redmond 1995; Spoth et al. 2000). Encouraging fathers to participate in parenting programs is important for several reasons. First, the time that fathers spend in childcare has increased dramatically over the past two decades, and many fathers want to be involved with their children (for review see Lamb 2010; Wells and Sarkadi 2012). There is also abundant evidence showing that fathers, much like mothers, have a substantial impact on children’s development and that father involvement is associated with positive child outcomes from infancy to adulthood (for review see Lamb 2010). Similarly, fathers’ use of ineffective parenting is related to child behavioral difficulties, just as mothers’ ineffective parenting is (Salari et al. 2014).

Data from the limited number of existing studies suggest that fathers’ participation in parenting programs is associated with better outcomes for children and may help to maintain treatment gains (see Lundahl et al. 2008). However, compared to mothers, fathers report fewer positive changes in their parenting or their child behavior (see Lundahl et al. 2008). There is also some evidence suggesting that fathers have a less positive perception of parenting programs and report less confidence in their ability to change their parenting behavior through participation in parenting programs (Niec et al. 2015; Tiano et al. 2013). Finally, fathers’ attendance in parenting programs is much lower than mothers’ (Panter-Brick et al. 2014). Thus, examining whether different factors impact mothers’ versus fathers’ participation in parenting programs will advance our understanding of how these programs can be more inclusive of fathers.

### Parenting Programs in Sweden

In Sweden, all forms of healthcare are free for children up to age 18. During the first year of a child’s life, child health services offer parental support to all parents, often in a form of parental groups with the primary aim of providing parents with a place to connect and establish a network. The availability of parenting support for parents of older children depends on local policies and competencies and varies greatly, with many municipalities offering no parenting programs at all. The national strategy encourages the implementation of evidence-based programs such as Positive Parenting Program (Triple P) and Incredible Years; however, offering locally developed programs is still more common. Preschools and schools are identified as two of the best places to reach parents. All preschools and schools—public or independent (charter)—are funded publically. For children 1 to 5 years old, preschools follow a maximum fee policy, capping the costs at about $150 per month (low-income families pay no fee). About 80 % of children in this age group attend preschool. For children 6 to 19 years old, schools have no entrance exam and are completely free.

There is no official record of the number of parents who participate in parenting programs. A recent study conducted in Sweden (Wells et al. 2016) reported that over a 6-month period, only 12 % of fathers attended a brief parenting program (three stand-alone large group seminars) offered universally to all parents of preschoolers as part of a research project. The corresponding rate for mothers was 23 %. This study also reported that participating parents, compared to non-participating parents, were more likely to be born in Sweden, have a university-level education, use more punitive parenting strategies, and experience higher levels of child difficulties. These findings are similar to the results from studies conducted in the USA and other western countries (e.g., Garvey et al. 2006).

### The Present Study

**Fig. 1:**
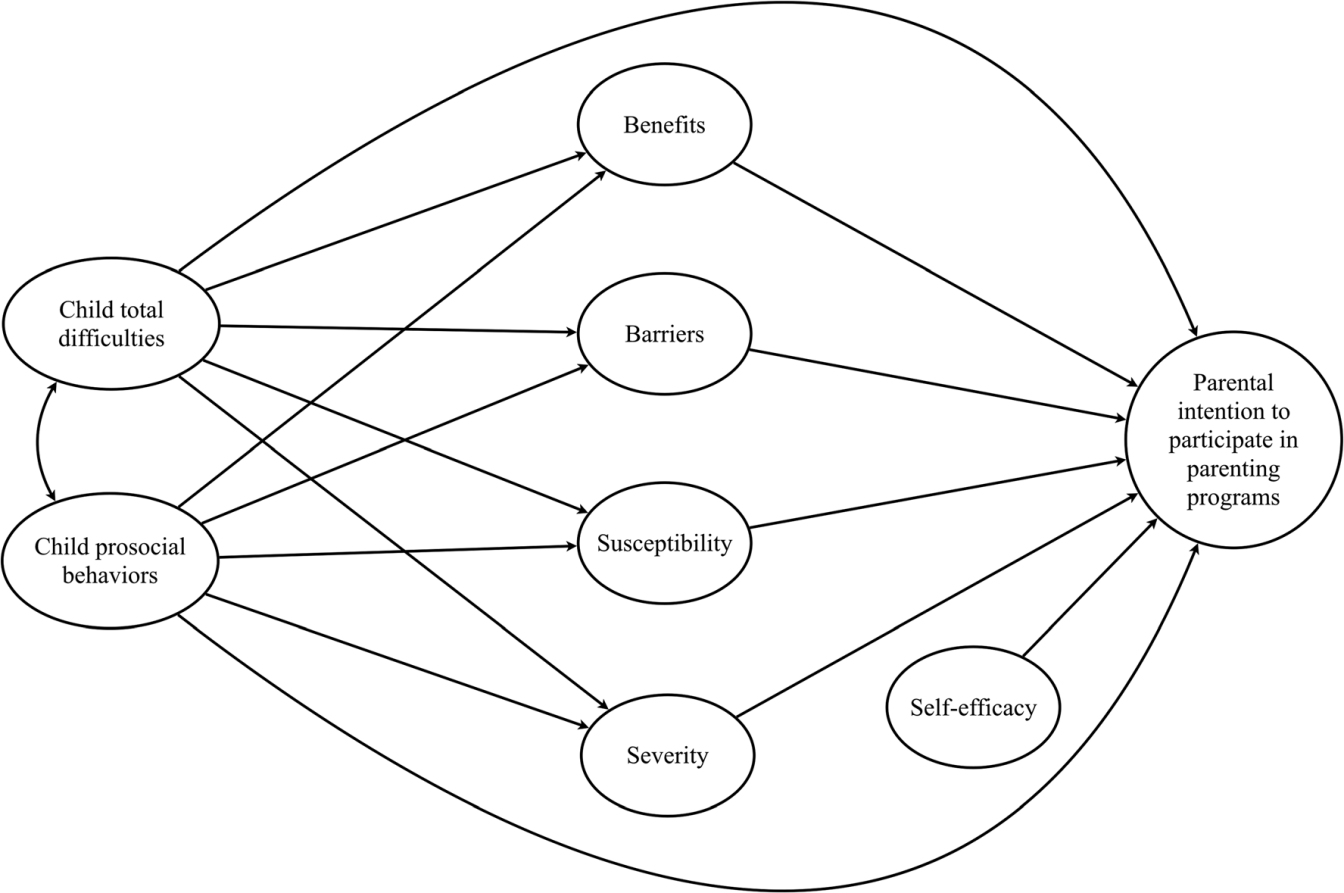
Hypothesized model of relations between the variables

In the present study, we addressed the limitations of previous research by using the HBM theoretical framework to examine factors, including the rarely studied factor of perceived self-efficacy, related to parents’ intention to enroll in parenting programs. In addition to the HBM variables, we also investigated the effects of child behavioral difficulties on parental intention to participate. Based on the studies mentioned earlier, we assumed that child problematic behaviors could influence parents’ attitudes directly and indirectly by affecting their perception of susceptibility, severity, benefits, and barriers. Finally, we investigated the possible moderating effects of parent gender on factors affecting parental intention to participate in parenting programs. Because this is a novel area of research, we did not have specific hypotheses regarding the moderating effects. The hypothesized model of relations between the variables is presented in Fig. 1.

## Method

### Participants and Procedure

Fifteen primary schools in Östersund and Strömsund (two small municipalities in central Sweden) participated in the study. Both mothers and fathers of all eligible children (children in preschool class and grades one to three) were contacted by e-mail and/or mail and invited to participate. All parents, regardless of whether they were originally contacted by e-mail and/or mail, were given the option to complete the questionnaire online or on paper.

Out of the 2340 mothers and fathers who were contacted, 795 (34.0 %) agreed to participate and later completed the survey. The vast majority of these parents (93.7 %) opted to complete the survey online. The sample consisted of 505 (63.5 %) mothers and 290 (36.5 %) fathers, representing 582 families (213 families were represented by both parents). To examine the moderating effects of parent gender, the sample size for mothers was matched with the fathers’ sample size by randomly selecting a subsample of 290 mothers out of the total of 505 mothers.

In the final sample, 122 families were represented by both parents. Mothers and fathers were more likely to be living together in the families represented by both parents compared to the families represented by one parent only (90.9 % compared to 79.2 %). The two groups of families did not differ on any other demographic variables (i.e., parental age, parental education, whether parents were born in Sweden, child age or child gender). The mothers’ age ranged from 24 to 52 years old (*M* = 39.14, SD = 5.04), and the fathers’ age ranged from 31 to 69 years old (*M* = 42.66, SD = 5.88). Only 4.7 % of the parents were born outside Sweden (other European countries, Middle East, East Asia, Africa, and South America). This is lower than the general population of 25- to 54-year-olds in Sweden (21.8 %) and in the two municipalities in this study (11.3 %). The parents in our sample were also more likely to have some form of post-high school education compared to the general population (51.2 % vs. 42.8 and 40.6 %, respectively). The children (51.7 % male) were between 5 to 10 years of age (*M* = 7.97, SD = 1.17), and the majority of them were living with both their biological parents (84.1 %).

### Measures

#### Child’s Problem Behavior

Parents’ perception of their child’s behaviors was assessed using the Strengths and Difficulties Questionnaire (SDQ; Goodman 1997), which is a measure of emotional and behavioral functioning in children. It has 25 items and 5 subscales: emotional symptoms, hyperactivity, conduct problems, peer problems, and prosocial behaviors. Each item is rated on a three-point scale from 0 (not true) to 2 (certainly true). The first four subscales are summed up to generate a total difficulties score. We used the official Swedish version of the measure which has been shown to have good internal consistency and discriminant validity (Malmberg et al. 2003). In the present study, the reliabilities for emotional symptoms, conduct problems, hyperactivity, and prosocial behaviors were satisfactory (*H* coefficients of 0.81, 0.74, 0.88, 0.72 and of 0.84, 0.78, 0.89, 0.77 for fathers and mothers, respectively). The reliability for peer problems was lower (*H* coefficients of 0.58 and 0.55 for fathers and mothers, respectively).

To measure the main constructs in the HBM model, we adapted the approach used by Spoth and Redmond (1995). The measures were developed in Swedish and were validated (in terms of factor structures and reliability) before conducting the main analyses (see the “Results” section and the Appendix, available online).

#### Perceived Benefits

The perceived benefits of participating in parenting programs were measured using 11 items evaluating the overall perceived benefits of participation. Each item described a possible benefit associated with participation in parenting programs (“if I attend a parenting program…”). Sample items included: “I can reduce the risk of my child developing problem behavior later in life” and “I can help my child be more self-confident.” Parents were asked to rate the degree to which they agreed with each statement on a four-point scale from completely agree (4) to completely disagree (1). Items were averaged to compute the total benefits score. The scale showed excellent reliability in this sample (*H* coefficients of 0.95 and 0.95 for fathers and mothers, respectively).

#### Perceived Barriers

We used eight items to measure parents’ perception of possible time-related (e.g., “I don’t have time”), psychological (e.g., “I’m worried about being criticized for how I am as a parent”), and logistic (e.g., “I don’t get support from my partner (my friends or my family) to attend a meeting”) obstacles that may prevent them from attending a parenting program. Parents rated each item on a four-point scale, ranging from strongly agree (4) to strongly disagree (1). Items were averaged to compute the scores for cognitive, logistic, and time-related barriers. The scale showed satisfactory reliability in this sample (*H* coefficients of 0.80, 0.76, 0.81 (fathers) and of 0.74, 0.83, 0.70 (mothers), for time-related, psychological, and logistic barriers, respectively).

#### Perceived Susceptibility

Parents’ perception of their children’s susceptibility was measured using 10 items evaluating child susceptibility to behavioral difficulties (e.g., “nagging,” “fighting with siblings or other children”) and performance difficulties (e.g., “giving up easily,” “having difficulties at school”). Each item described a mild behavioral issue that is addressed in universal parenting programs, in this case All Children in Focus (the ABC program; Ulfsdotter et al. 2014), which was available to the parents in this sample free of charge. Parents were asked to indicate how often they thought their child would engage in each behavior 2 years from now. Responses to each item were given on a four-point scale, ranging from never (1) to often (4). Items were averaged to compute the total scores on susceptibility to behavioral and performance difficulties. The scale showed satisfactory reliability (*H* coefficients of 0.83, 0.83 (fathers) and of 0.84, 0.81 (mothers) for the behavioral and performance difficulties, respectively).

#### Perceived Severity

Parents’ perception of the undesirable impact of their children’s behavioral problems and performance difficulties was measured using the same 10 items used for perceived susceptibility. Parents were asked to indicate how negative it would be if their child were to engage in each behavior 2 years from now. Each item was rated on a four-point scale, ranging from not negative at all (1) to very negative (4). Items were averaged to compute the total scores on severity of behavioral and performance difficulties. The scale showed excellent reliability in this sample (*H* coefficients of 0.76, 0.87 (fathers) and of 0.86, 0.88 (mothers) for the behavioral and performance difficulties, respectively).

#### Perceived Self-Efficacy

Perceived self-efficacy to acquire new parenting knowledge and skills was measured using 10 items assessing parents’ confidence in their ability to learn, discuss, and implement new information and strategies. Sample items included: “I can learn a lot by listening to lectures” and “I can change the way I behave with my child.” Items were rated on a four-point scale, ranging from completely agree (4) to completely disagree (1). Items were averaged to yield a total self-efficacy score. The scale showed excellent reliability in this sample (*H* coefficients of 0.90 and 0.90 for fathers and mothers, respectively).

#### Intention to Participate

Intention to participate in parenting programs was measured by asking parents to indicate how likely they would be to participate in four different modules of parenting programs: web based, seminar, group, and individual. Each format was rated on a four-point scale, from very likely (4) to not at all likely (1). Items were averaged to yield an overall indicator of parental intention to participate. This indicator was treated as a formative construct (Fornell and Bookstein 1982), for which the measures of internal consistency are not relevant.

### Data Analysis Strategy

#### Measurement Equivalence

In the first step, using multi-group confirmatory factor analysis (CFA) in Mplus v. 7.3 (Muthén and Muthén 1998–2012), we evaluated the equivalence of measures between mothers and fathers to establish whether meaningful comparisons could be made between the two genders. Equivalence is a term that describes the different aspects of comparability of the constructs across two or more groups (Byrne and Watkins 2003).

#### Scale Reliability

Due to the limitations associated with Cronbach’s alpha coefficient when the assumptions of tau-equivalence and/or uncorrelated errors are violated, we assessed the internal consistency by calculating the *H* coefficient (Hancock and Mueller 2001). The range and interpretations of the *H* coefficient are exactly the same as for Cronbach’s alpha.

#### Evaluating Relations Between Variables

First, we investigated the correlations between all the constructs of interest at the latent level in Mplus v. 7.3 (Muthén and Muthén 1998–2012). The latent approach allows for an estimation of effect sizes that are not attenuated by measurement error (Kline 2011). Next, the hypothesized model and moderating effects of parent gender were evaluated via multi-group structural equation modeling (SEM) approach, also in Mplus.

#### Model Estimation and Evaluation

The factor structures of all the measures (part of the equivalence testing procedure) were estimated using the mean- and variance-adjusted weighted least square estimator (WLSMV), given the ordinal nature of all the observed indicators (four-point Likert scale) (Muthén et al. 1997). For the correlation analysis and the evaluation of the hypothesized model, we applied the robust maximum likelihood (MLR) estimator for continuous indicators (see below) to account for the multivariate non-normality of the data (see the “Data Screening” section).

For the correlation analysis and SEM, the scales’ indicators were grouped into parcels to control for inflated measurement errors and improve the psychometric properties of the variables (Little et al. 2002). Each parcel represented an average of the items included therein. This allowed us to treat the observed indicators (parcels) as continuous. For the dependent variable, measured using just one observable indicator, a single-indicator latent construct was created using the guidelines proposed by Speirs and Martin (1999). The chi-square (*χ* ^2^) goodness-of-fit statistic, the comparative fit index (CFI), the root mean square error of approximation (RMSEA) with 90 % CI, and the standardized root mean square residual (SRMR; only available for MLR estimator) were used to evaluate each model fit. For the model to be considered to have acceptable fit, RMSEA and SRMR should be <0.08 with CFI >0.90 (Hu and Bentler 1999). Models were re-specified based on modification indices (MIs), inspection of standardized residuals, and theoretical considerations (Kline 2011). To assess the extent to which the newly specified model exhibited an improvement over its predecessor, we used different approaches suitable for the two estimators. For the WLSMV estimator, the chi-square difference test was calculated using the *difftest* command in Mplus (Muthén and Muthén 1998–2012). For the MLR, the chi-square difference test was applied for nested models (using the scaled chi-square and formulas by Satorra and Bentler 1994), as well as the Akaike information criterion (AIC) and the Bayesian information criterion (BIC) values for non-nested models (Schreiber et al. 2006). Finally, to test the significance of the hypothesized mediation effects, the bootstrap method with 5000 bootstrap samples was used (Shrout and Bolger 2002).

## Results

### Data Screening

The percentage of missing data points was 0.03. The Little’s missing completely at random (MCAR) test indicated that the data were missing completely at random, MCAR [*χ* ^2^(2063) = 2086.96, *p* = .35]. Two methods were applied to appropriately handle missing data (relevant for the two estimators used). For the WLSMV estimator (used for CFAs), we used the *pairwise present method*, which uses polychromic correlations for pairwise present data, where the WLSMV ignores only the missing values involved in the two variables, as opposed to all of the information about the case (Asparouhov and Muthén 2010). For the MLR estimator (correlation analysis and SEM), we applied the full information maximum likelihood (FIML) approach (Enders 2001). Both methods yield unbiased results under the MCAR assumption. In terms of distribution assumptions, the data showed significant multivariate skew (1584.05, *χ* ^2^ = 119,859, *p* < .001) and kurtosis (7205.46, *Z* = 44.32, *p* < .001). Furthermore, squared Mahalanobis distances (*D* ^2^) indicated 10 serious multivariate outliers, which were deleted from further analysis, giving the final sample of *N* = 570 (286 mothers and 284 fathers).

Since (a) the participants were recruited from different schools and (b) there were 122 couples in the data, the clustered nature of the data had to be considered. A substantial degree of non-independence in the data would require the application of multi-level modeling to account for non-independence. The degree of non-independence in the data was assessed using interclass correlation coefficients (ICCs), where values >0.05 indicate that non-independence is too high to be ignored (Hox 2010). For the current data set, the ICC values for all the observed variables were <0.05, indicating no need for a multi-level modeling approach.

### Measurement Equivalence and Scale Reliability

The equivalence of measures across mothers and fathers was assessed in three common steps (Byrne and Watkins 2003): (a) test of configural invariance (the same factorial structures across the groups), (b) test of metric invariance (equal factor loadings across the groups), and (c) test of scalar invariance (equal intercepts across the groups). For the present study, it was particularly vital to establish metric equivalence for all the measures, as it represents the necessary condition for conducting valid group comparisons on the relations between the constructs. The analyses were lengthy, and describing them in detail is beyond the scope of the present paper (they are included in the Appendix, available online). The analyses provided support for the metric equivalence between mothers and fathers for all the measures, which allowed us to evaluate the moderating effects of parent gender.

### Analysis of the Factors Affecting Intention to Participate in Parenting Programs

The results of the correlation analysis (see Table 1) suggested that parent gender may have moderating effects on the hypothesized direct and indirect relations between the constructs. For both fathers and mothers, perceived child emotional and behavioral problems were positively and significantly associated with perceived benefits, barriers, and susceptibility (Table 2). Also for both mothers and fathers, perceived child prosocial behaviors were significantly and negatively related to perceived susceptibility. However, only for fathers were perceived child prosocial behaviors positively related to perceived severity. Furthermore, for both mothers and fathers, perceived child emotional and behavioral problems and benefits were positively associated with parental intention to participate in parenting programs. However, for fathers, perceived self-efficacy was also significantly and positively associated with intention to participate.

**Table 1:** Pearson product-moment correlations among child adjustment (SDQ); perceived benefits, barriers, susceptibility, severity, and self-efficacy; and intention to participate in parenting programs for mothers and fathers

	1	2	3	4	5	6	7	8	Mean	SD
1. SDQ total difficulties		−0.50***	0.19*	0.49***	−0.24*	0.60***	0.16	0.27***	5.17	4.01
2. SDQ prosocial behaviors	−.56***		0.06	−0.10	0.15	−0.30***	−0.08	−0.11	4.83	1.21
3. Benefits	0.24**	0.01		0.19*	0.14	0.12	0.15	0.38***	2.96	0.69
4. Barriers	0.33**	−0.12	0.09		−0.64***	0.23*	0.33***	−0.13	1.75	0.46
5. Self-efficacy	0.01	0.19*	0.41***	−0.40***		−0.12	−0.14	0.15	3.51	0.39
6. Susceptibility	0.66**	−0.42***	0.15	0.14	0.03		−0.01	0.11	2.49	0.41
7. Severity	0.03	0.21**	0.22**	0.12	0.16	−0.08		0.08	2.82	0.49
8. Intention to participate	0.18*	−0.04	0.53***	−0.09	0.44***	0.11	0.07		2.69	0.62
Mean	6.07	4.36	2.93	1.75	3.24	2.45	2.87	2.41		
SD	4.13	1.31	0.64	0.44	0.47	0.40	0.42	0.64		

To evaluate correlations at the latent level, we used MLR estimator (Pearson correlations) and FIML procedure to handle missing data. Coefficients above the diagonal pertain to mothers, and coefficients below the diagonal pertain to fathers. Means and SDs in the last two columns on the right hand side pertain to mothers, and means and SDs in the last two bottom rows pertain to fathers. Because the metric equivalence for mothers and fathers was not supported for most of the scales (see Appendix, available online), testing for mean differences between mothers and fathers is warranted (Byrne and Watkins 2003)

*SDQ* Strengths and Difficulties Questionnaire

*p < .05; **p < .01; ***p < .001

**Table 2:** Assessment of the model testing the relations between child adjustment (SDQ); perceived barriers, benefits, susceptibility, severity, and self-efficacy; and parental intention to participate in parenting programs

Model	*χ* ^2^	*df*	Δ*χ* ^2^	Δ*df*	CFI	SRMR	RMSEA	RMSEA 90 % CI
*Model for fathers* With added correlations between the error terms of parcels 1 and 4 of the SDQ total difficulties and of parcels 1 and 4 of perceived susceptibility	365.41***	253			0.951	0.056	0.039	0.030–0.048
*Model for mothers* With added correlations between the error terms of parcels 1 and 4 of the SDQ total difficulties, parcels 1 and 4 of perceived benefits, and of parcels 1 and 4 of perceived susceptibility	412.52***	252			0.921	0.060	0.047	0.039–0.055
*Free estimated model*	777.95***	502			0.936	0.058	0.044	0.037–0.049
*Measurement invariance* All factor loadings constrained equal except from parcel 1 of perceived self-efficacy and parcel 1 of perceived severity	812.01***	524	34.07^a^	22	0.933	0.070	0.044	0.038–0.049
*Invariance of structural paths* All factor loadings constrained equal except from parcel 1 of perceived self-efficacy and parcel 1 of perceived severity, and all paths constrained equal except from paths from total difficulties, perceived barriers, and self-efficacy to intention to participate	819.97***	536	43.81^a^	34	0.934	0.070	0.043	0.037–0.048

All models based on *N* = 284 for fathers and *N* = 286 for mothers

*χ* ^*2*^ chi-square, *df* degrees of freedom, *CFI* comparative fit index, *SRMR* standardized root mean square residual, *RMSEA* root mean square error of approximation, *CI* confidence interval, *SDQ* Strengths and Difficulties Questionnaire

***p < .001

^a^As compared with the free estimated model

**Fig. 2:**
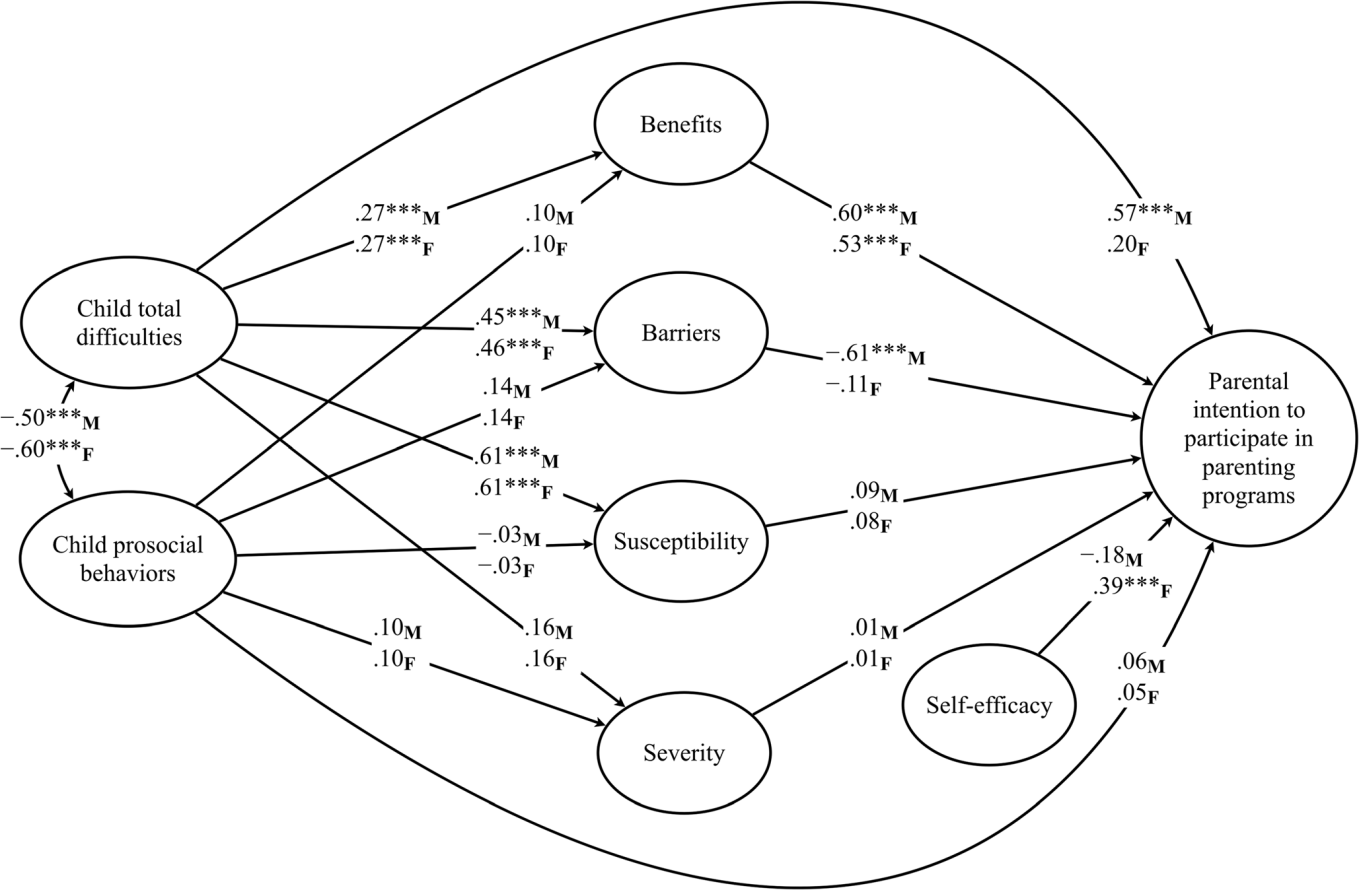
Structural equation model of predictors of parental intention to participate in parenting programs. Standardized estimates for fathers and mothers. Model fit *χ* ^2^ (536) = 819.97, *p* < .001; CFI = 0.934; RMSEA = 0.043; 95 % CI (0.037–0.048); SRMR = 0.070; Model based on *N* = 284 fathers and *N* = 286 mothers; all factor loadings significant at *p* < .001; *F* fathers, *M* mothers. ***p < .001

In the next step, the hypothesized model was evaluated (see Table 3 for the overview of the analyses). The final model showed good fit to the data (see Table 3). A simple graphic representation of the model is presented in Fig. 2 (the full model is available from the first author). The analyses revealed equivalence between mothers and fathers in terms of the effects of perceived child emotional and behavioral problems, as well as prosocial behaviors, on perceived benefits, barriers, susceptibility, and severity. For both mothers and fathers, the higher were perceived child problem behaviors, the more benefits, barriers, and child susceptibility the parents perceived. Child emotional and behavioral problems had no effect on perceived severity. No significant effects of child prosocial behaviors on perceived benefits, barriers, susceptibility, and severity were found. However, the analyses revealed moderating effects of parent gender on the direct effects of perceived benefits, barriers, susceptibility, severity, and self-efficacy on parental intention to participate in parenting programs. For mothers, higher perceived benefits and lower perceived barriers were associated with stronger intention to participate. For fathers, higher perceived benefits and higher self-efficacy were associated with stronger intention to participate.

**Table 3:** Total, simple indirect, and total indirect effects for the relations between child adjustment (SDQ); perceived barriers, benefits, susceptibility, severity, self-efficacy; and parental intention to participate in parenting programs, unstandardized estimates

Simple indirect effect	Estimate	95 % bootstrap CI	Estimate	95 % bootstrap CI
	Fathers		Mothers	
Total effects				
SDQ total difficulties → intention to participate	0.106	−0.014–0.889	0.123	−0.126–0.260
SDQ prosocial behaviors → benefits → intention to participate	0.032	−0.060–0.853	0.010	−0.100–0.237
Simple indirect effects				
SDQ total difficulties → benefits → intention to participate	**0.050**	**0.015**–**0.235**	**0.050**	**0.015**–0**.235**
SDQ total difficulties → barriers → intention to participate	−0.017	−0.771–0.050	−0.086	−1.733–0.007
SDQ total difficulties → susceptibility → intention to participate	−0.017	−0.079–0.037	−0.017	−0.079–0.037
SDQ total difficulties → susceptibility → intention to participate	0.000	−0.163–0.013	0.000	−0.163–0.013
SDQ prosocial behaviors → benefits → intention to participate	0.019	−0.016–0.193	0.019	−0.016–0.193
SDQ prosocial behaviors → barriers → intention to participate	−0.005	−0.626–0.021	−0.027	−1.195–0.054
SDQ prosocial behaviors → susceptibility → intention to participate	0.001	−0.014–0.015	0.001	−0.014–0.015
SDQ prosocial behaviors → susceptibility → intention to participate	0.000	−0.154–0.012	0.000	−0.154–0.012
Total indirect effects				
SDQ total difficulties → benefits → intention to participate	0.015	−0.820–0.110	−0.054	−1.711–0.071
SDQ prosocial behavior → benefits → intention to participate	0.014	−0.612–0.071	−0.008	−1.358–0.084

*CI* confidence interval, significant effects in bold. *SDQ* Strengths and Difficulties Questionnaire

The analyses of the mediation effects (see Table 3) revealed no moderating effects of parent gender. For both mothers and fathers, the effect of perceived child emotional and behavioral difficulties on parental intention to participate was mediated by perceived benefits. The combined set of predictors explained 45 and 46 % of the variance in paternal and maternal intention to participate, respectively.

## Discussion

The present study evaluated a comprehensive model of factors affecting parental intention to participate in parenting programs. The model was based on the Health Belief Model theoretical framework and included perceived self-efficacy to learn and implement new parenting strategies. More importantly, our approach was unique in that we explored the moderating effects of parent gender. With regard to the HBM model, our findings are consistent with previous studies (Spoth and Redmond 1995; Spoth et al. 2000; Thornton and Calam 2011) showing that perceived benefits were the strongest predictor of parental intention to attend parenting programs, followed by perceived barriers, while perceived child susceptibility and severity had no or only a negligible relation to parental intention to participate. Our findings are also in line with earlier studies indicating that parents who report higher levels of difficulties in their children are more likely to show an interest in parenting programs (Thornton and Calam 2011) and that this is mainly because parents of more difficult children perceive parenting programs as more beneficial (Spoth et al. 2000). None of these studies, however, have tested the moderating effects of parents’ gender or included measures of perceived self-efficacy to learn and implement new parenting strategies.

While enrollment and attendance rates are substantially lower for fathers compared to mothers, most of our knowledge about program engagement comes from studies on samples that are composed only or mainly of mothers (Panter-Brick et al. 2014). Research indicates that it is important to encourage fathers’ involvement in parenting programs because (a) fathers’ use of ineffective parenting strategies is related to child behavior problems, just like mothers’ use of ineffective parenting strategies (e.g., Salari et al. 2014), and (b) fathers’ participation in parenting programs may help improve child outcomes as well as maintain the intervention gains over time (see Lundahl et al. 2008; Panter-Brick et al. 2014). Increasing fathers’ engagement requires a greater understanding of the factors that affect their decision to participate in parenting programs. Our study is one of the first to evaluate the factors affecting intention to participate in parenting programs for both mothers and fathers.

The present study found that, for both mothers and fathers, higher perceived benefits were associated with higher intention to participate in parenting programs. In addition, for both mothers and fathers, perceived child emotional and behavioral problems had an indirect effect on parents’ intention to participate by increasing the level of perceived benefits of the program. These comparable results for mothers and fathers may be due to the similarities between how mothers’ and fathers’ perceptions of their own parenting are related to their ratings of behavior problems in their children. For example, it has been shown that, much like mothers, fathers who report using more punitive parenting strategies tend to rate their children as having more behavioral difficulties (Salari et al. 2014).

The present findings also showed that fewer perceived barriers predicted higher intention to participate for mothers, but not for fathers, while higher perceived self-efficacy predicted higher intention to participate for fathers, but not for mothers. One reason for these disparate findings may be the differences in maternal and paternal roles in relation to children. While based on political, educational, and economic indicators (e.g., political representation, workforce participation, and wage), Sweden is recognized as one of the world’s most gender-equal countries, Swedish mothers still assume more responsibility for taking care of their children than Swedish fathers (Statistics Sweden 2014). Health professionals also tend to focus on mothers and treat fathers as secondary parents (Wells and Sarkadi 2012). Therefore, as the primary caregivers, mothers may feel more responsible for engaging in programs that concern their children, even when they believe that they are not likely to learn and implement what is discussed during the program. However, logistic and psychological barriers may severely limit their ability to engage in these programs. In contrast, fathers may not feel obliged to consider attending a program when they have less confidence in their ability to learn new skills or change their own behavior. However, logistic and psychological barriers may not affect their intention to participate, as they may perceive themselves as capable of overcoming these barriers if necessary.

The disparate findings for perceived barriers may also be due to the types of barriers assessed in this study. These barriers may be more relevant for mothers than for fathers. While barriers such as lack of time may be more relevant for mothers, work-related barriers (e.g., lack of organizational support) or program-specific features (e.g., composition of participants) may have a greater impact on fathers’ intention to participate in parenting programs (Bayley et al. 2009). Another explanation is that mothers may simply have a more realistic estimation of their ability to attend parenting programs. Similarly, it should also be noted that while outcome expectancies such as perceived benefits and barriers seem to play a predominant role in forming the intention to take action, self-efficacy beliefs are considered to be important in both initiation and maintenance of an action (Schwarzer and Fuchs 1995). In the current study, mothers’ perceived self-efficacy was not related to their intention to participate in parenting programs. Nonetheless, their self-efficacy may be related to their actual attendance or whether their attendance leads to any real change in their parenting behavior or beliefs. Future studies should examine these issues further in order to even better inform the development of effective engagement strategies.

Gender equality is highly institutionalized in Sweden, and fathers are encouraged and expected to take an active role in caring for their children from very early on (although they are still viewed and treated as secondary caregivers). Our findings showed that even in gender-equalitarian Sweden, the factors affecting participation in parenting programs may be different for mothers and fathers. Thus, we speculate that there may be even greater differences in countries with more gendered maternal and paternal roles. This finding stresses the need for researchers to collect data from both mothers and fathers and to assess the moderating effects of parent gender in their analyses. This approach is just as important in studies of program participation and engagement as it is in studies of program effectiveness and efficacy. In addition, researchers should be more vigilant when selecting and conceptualizing the factors they will examine to ensure that future studies on parental participation include factors that are relevant for fathers as well. Our findings also indicate that persuading fathers to participate in parenting programs requires making a dedicated effort to target them more specifically. In their recent paper, Thornton and Calam (2011) discussed how parents’ perception of parenting programs can be changed to increase participation in these programs. They suggested that promotional materials such as leaflets, flyers, and posters should incorporate relevant program information and present this information using an attractive design. In light of our findings, we argue that when preparing promotional materials, we also need to be aware that these materials should communicate effectively to both mothers and fathers. Employing a Socratic argument about the benefits of parenting programs may be useful in attracting both mothers and fathers. However, the type of benefits highlighted may need to be different (Wells et al. 2016). In addition, fathers’ self-efficacy beliefs about their ability to change their behavior should be targeted specifically. For example, flyers can include statements from fathers who have previously attended the program and found it easy to implement the strategies at home.

Some of the present study’s limitations should be noted. First, similar to other studies, we asked parents to focus on one of their children when completing the questionnaires. Most parents have more than one child, and their decision about whether to participate in a program may not be entirely based on the characteristics of the child in focus. Second, our findings cannot necessarily be generalized to mothers and fathers of children with clinically elevated behavioral and emotional problems, as our sample was drawn from a general population. Moreover, our sample consisted of mothers and fathers who, compared to the general Swedish population, were more likely to be born in Sweden and have a university-level education. However, highly educated parents from a Swedish background are more likely to conform with gender-equity values (Edlund and Öun, 2016); therefore, one can expect the differences found in this study to be greater in populations with more gendered parental roles. Finally, we investigated predictors of parental intention to participate, not actual participation. Research indicates that there is a discrepancy between expressing interest in participating in a program (inclination/intention) and actual participation (Spoth et al. 1997). Although intention to enroll predicts actual attendance (Bauman et al. 2001; Díaz et al. 2006; Spoth et al. 2007), when parents indicate they intend to attend a program, it does not necessarily mean they will actually do so. Therefore, future studies should examine whether the same patterns of association are observed for mothers’ and fathers’ actual participation in parenting programs.

## Electronic supplementary material

ESM 1 (PDF 423 kb)
