# Supplementary material for: Using the Health Belief Model to Explain Mothers’ and Fathers’ Intention to Participate in Universal Parenting Programs
Source: Prev Sci. 2016 Sep 17;18(1):83–94. doi: 10.1007/s11121-016-0696-6 (PMC5236081; doi:10.1007/s11121-016-0696-6)
Supplement: Supplementary file 1 — (PDF 423 kb) [file 11121_2016_696_MOESM1_ESM.pdf]

Appendix

Analyses of Measurement Equivalence

Table A1

Evaluation of measurement invariance assessment for all measures included in the study

Model	WLSMVχ^2	*df*	$\Delta\chi^2$	Δdf	CFI	RMSEA	RMSEA 90% CI
The SDQ							
Configural invariance 5-factor, 17-item congeneric model	452.95***	219			.920	.061	.053 – .069
Metric invariance All factor loadings constrained equal except for loadings of items 2 & 24	457.88***	234	19.13[a]	15	.924	.057	.050 – .065
Scalar invariance [c] All factor loadings and intercepts constrained equal except for intercepts from items 2 & 24	484.51***	248	36.33***[b]	14	.920	.057	.050 – .065
The perceived benefits scale							
Configural invariance 1-factor, 11-item congeneric model	452.62***	99			.978	.111	.101 – .122
Metric invariance All factor loadings constrained equal	308.43***	110	11.75[a]	11	.988	.079	.069 – .090
Scalar invariance [c] All factor loadings and intercepts constrained equal except for intercepts from items 6, 10, & 11	307.74***	118	14.79[b]	8	.988	.075	.064 – .085
The perceived barriers scale							
Configural invariance 3-factor, 7-item congeneric model (item 5 removed)	53.08***	29			.973	.054	.030 – .076
Metric invariance All factor loadings constrained equal	55.25***	36	7.08[a]	7	.978	.043	.017 – .064
Scalar invariance All intercepts constrained equal	59.01***	43	5.75[b]	7	.982	.036	.000 – .057

Table A1 (continued)

Model	WLSMVχ^2	*df*	$\Delta\chi^2$	Δdf	CFI	RMSEA	RMSEA 90% CI
The perceived severity scale							
Configural invariance 1-factor, (9-item congeneric model (with item 5 removed)	125.02***	61			.976	.060	.045 – .075
Metric invariance All factor loadings constrained equal except for items 1, 2, 3, & 8	128.27***	66	9.52[a]	5	.977	.057	.042 – .072
Scalar invariance [c] All intercepts constrained equal except intercepts for items 1, 2, 3, & 8)	130.37***	71	6.02[b]	5	.978	.054	.039 – .068
The perceived susceptibility scale							
Configural invariance 1-factor, (9-item congeneric model (with item 5 removed)	135.74***	61			.971	.066	.051 - .081
Metric invariance All factor loadings constrained equal except for items 1, 2, 3, & 8	124.19***	69	5.43[a]	9	.977	.053	.037 – .067
Scalar invariance [c] All intercepts constrained equal except intercepts for items 1, 2, 3, & 8)	126.05***	74	4.9[b]	5	.980	.049	.034 – .064
The self-efficacy scale							
Configural invariance 1-factor, (7-item congeneric model (with items 1, 4, & 9 removed)	71.72***	31			.980	.067	.047 – .088
Metric invariance All factor loadings constrained equal except for items 2, 6, & 7	74.86***	35	8.74[a]	4	.981	.063	.043 – .082
Scalar invariance [c] All intercepts constrained equal except the intercepts for items 2, 6, & 7	147.79***	39	61.19***[b]	4	.948	.098	.082 – .115

All models based on $N = 570$ ($N = 284$ fathers and $N = 286$ mothers)
WLSMV χ^2 = chi-square statistic from the robust weighted least squares estimator, *df* = degrees of freedom, CFI = comparative fit index, RMSEA = root mean square error of approximation, CI = confidence interval, SDQ = Strengths and Difficulties Questionnaire
***$p < .001$
[a] as compared with the free estimated model
[b] as compared with the metric equivalence model
[c] subsequent tests did not provide support for partial scalar invariance

Summary of the results presented in Table A1

The SDQ The analysis indicated that the 5-factor, 17-item model fitted the data satisfactorily for both mothers and fathers. Eight items needed to be removed from the original model due to insignificant factor loadings or lack of associations with other items.[1] The results supported partial metric invariance across mothers and fathers.[2]

The perceived benefits scale The analysis indicated that the 1-factor, 11-item model showed satisfactory fit for both mothers and fathers. The results supported full metric and partial scalar invariance across mothers and fathers.

The perceived barriers scale The analysis indicated that the 3-factor, 7-item model fitted the data well for both fathers and mothers. One item needed to be removed from the model, due to insignificant factor loading.[1] The results supported full metric and scalar invariance across mothers and fathers.

The perceived severity scale The CFA revealed that the 2-factor, 9-item model fitted the data well for both fathers and mothers. One item needed to be removed from the model due to insignificant factor loadings.[1] The results supported full metric and scalar invariance across mothers and fathers.

The perceived susceptibility scale The analysis showed that the 2-factor, 9-item model fitted the data well for both fathers and mothers. One item needed to be removed from the model due to insignificant factor loadings.[1] The results supported full metric and scalar invariance across mothers and fathers.

The perceived self-efficacy scale The analysis indicated that the 1-factor, 7-item model fitted the data well for both groups. Three items needed to be removed for the models due to insignificant factor loadings. The results supported full metric and scalar invariance across mothers and fathers.

[1] For details, please contact the corresponding author.
[2] Recent literature indicates that valid comparisons can be made if only partial equivalence is achieved (at least two indicators per latent variable are equivalent between groups; Byrne, B. M., & Watkins, D. (2003). The issue of measurement invariance revisited. *Journal of Cross-Cultural Psychology, 34*(2), 155-175. doi: 10.1177/0022022102250225).

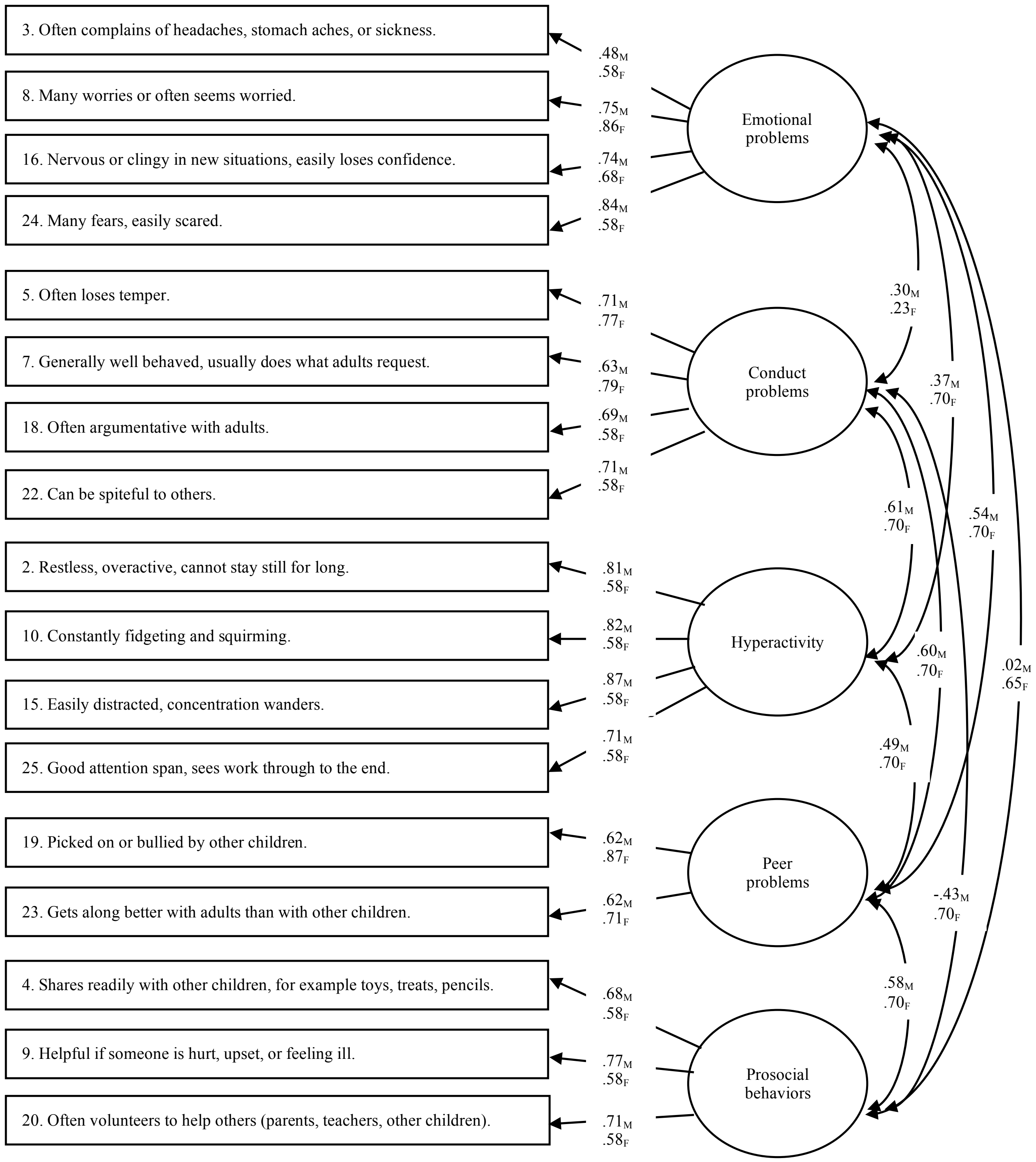

Fig. A1

Factor structure of the SDQ (Strengths and Difficulties Questionnaires) for mothers and fathers. Standardized factor loadings. Model fit: WLSMV χ^2 (234) = 457.88, $p < .001$; CFI = .924; RMSEA = .057 95%CI (.050-.065); all models based on N=284 fathers and N = 286 mothers; all factor loadings significant at $p < .001$

How much do you agree with each of the following statements?
If I attend a parenting program…

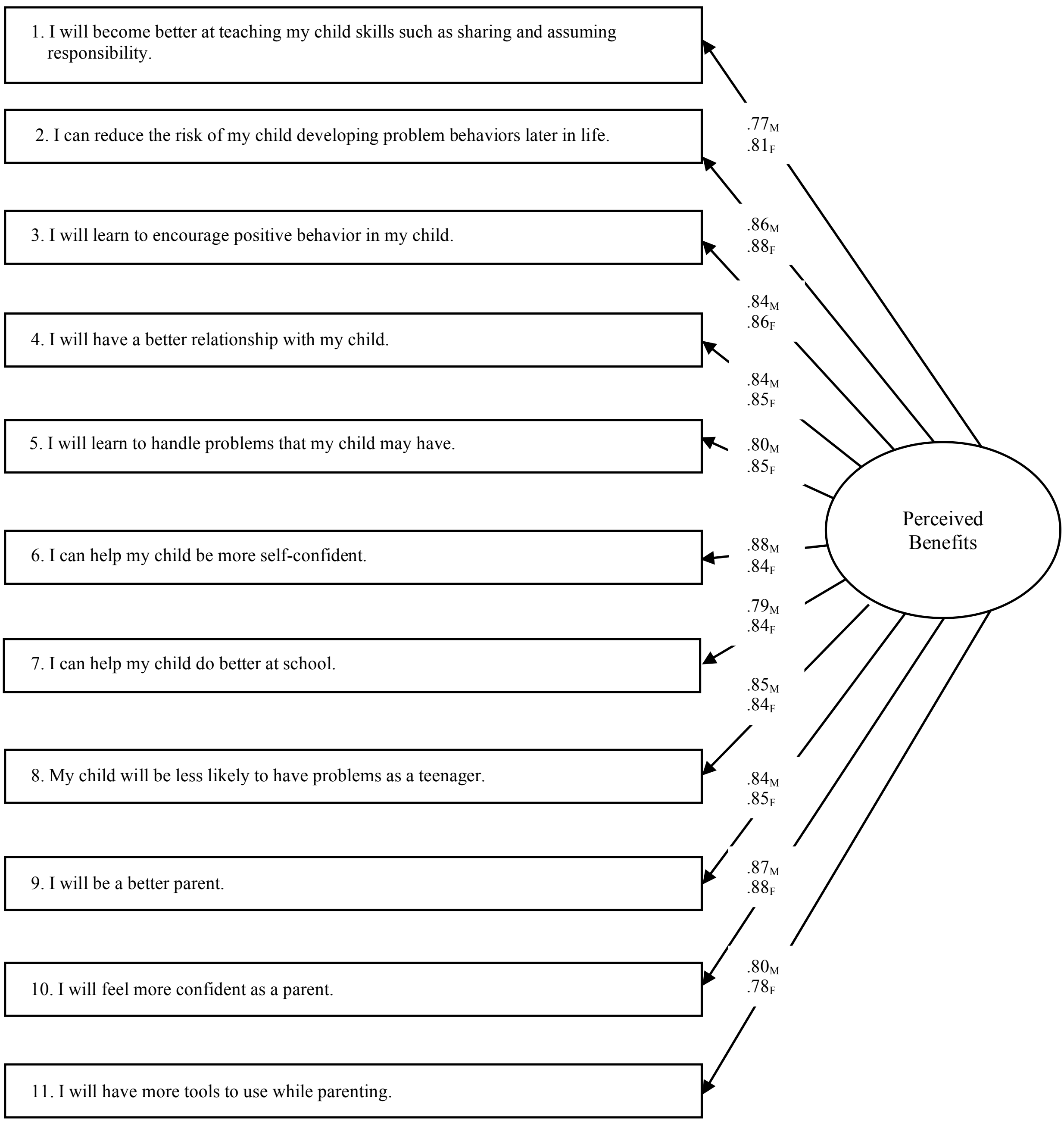

Fig. A2

Factor structure of the perceived benefits scale for mothers and fathers. Standardized factor loadings. Model fit: WLSMV χ^2 (118) = 307.74, $p < .001$; CFI = .988; RMSEA = .075 95%CI (.064 – .085); all models based on N=284 fathers and N = 286 mothers; all factor loadings significant at $p < .001$

There are many reasons why parents might not attend a parenting program. Below is a list of some common ones. For each item, please indicate how much it would stop you from attending.

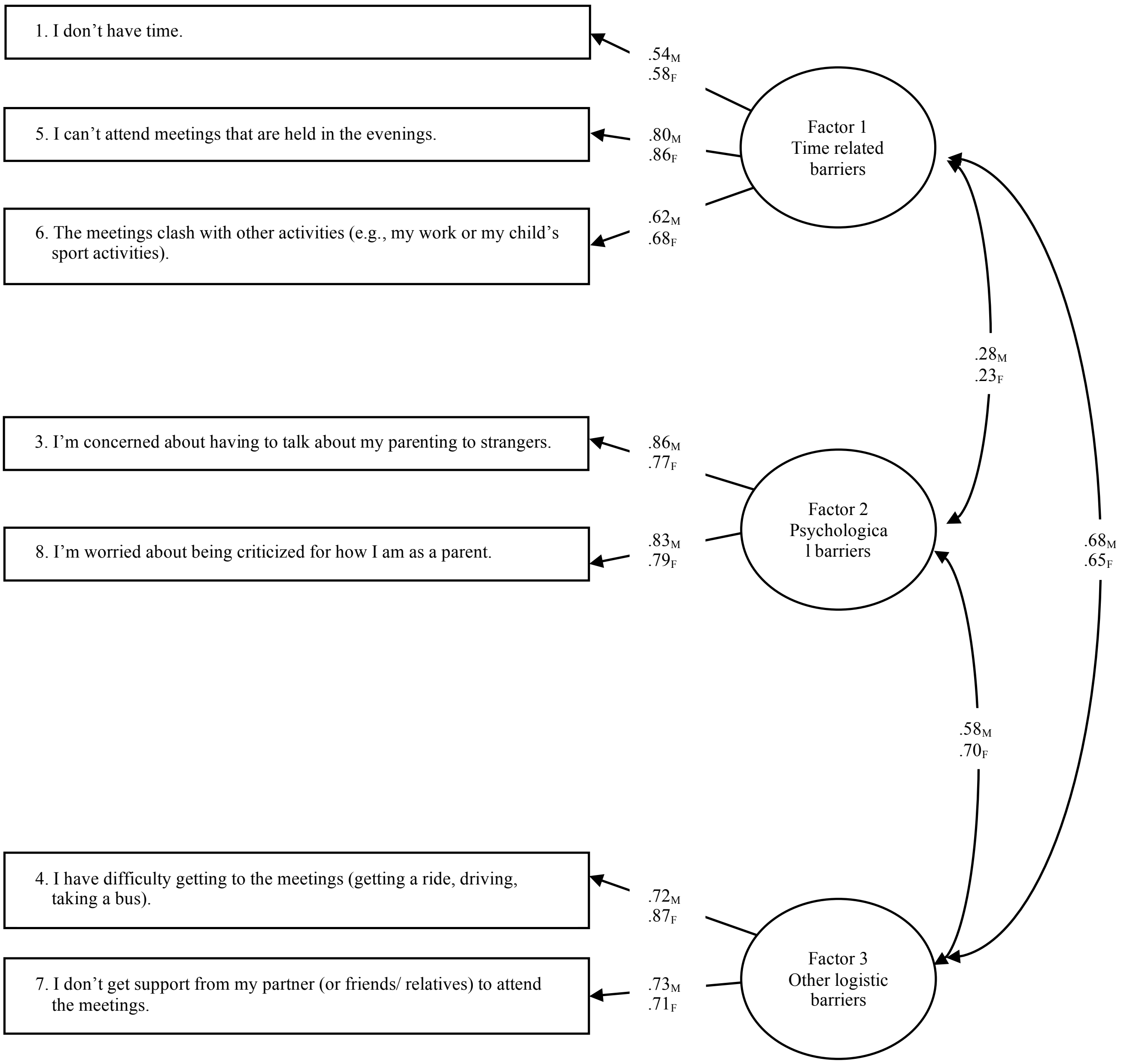

Fig. A3

Factor structure of the perceived barriers scale for mothers and fathers. Standardized factor loadings. Model fit: WLSMV χ^2 (43) = 59.01, p < .001; CFI = .982; RMSEA = .036 95%CI (.000 – .057); all models based on N=284 fathers and N = 286 mothers; all factor loadings significant at p < .001; correlation between latent constructs significant at p < .001

How often do you think your child will engage in (/experience) each of the following behaviors (/difficulties) two years from now?

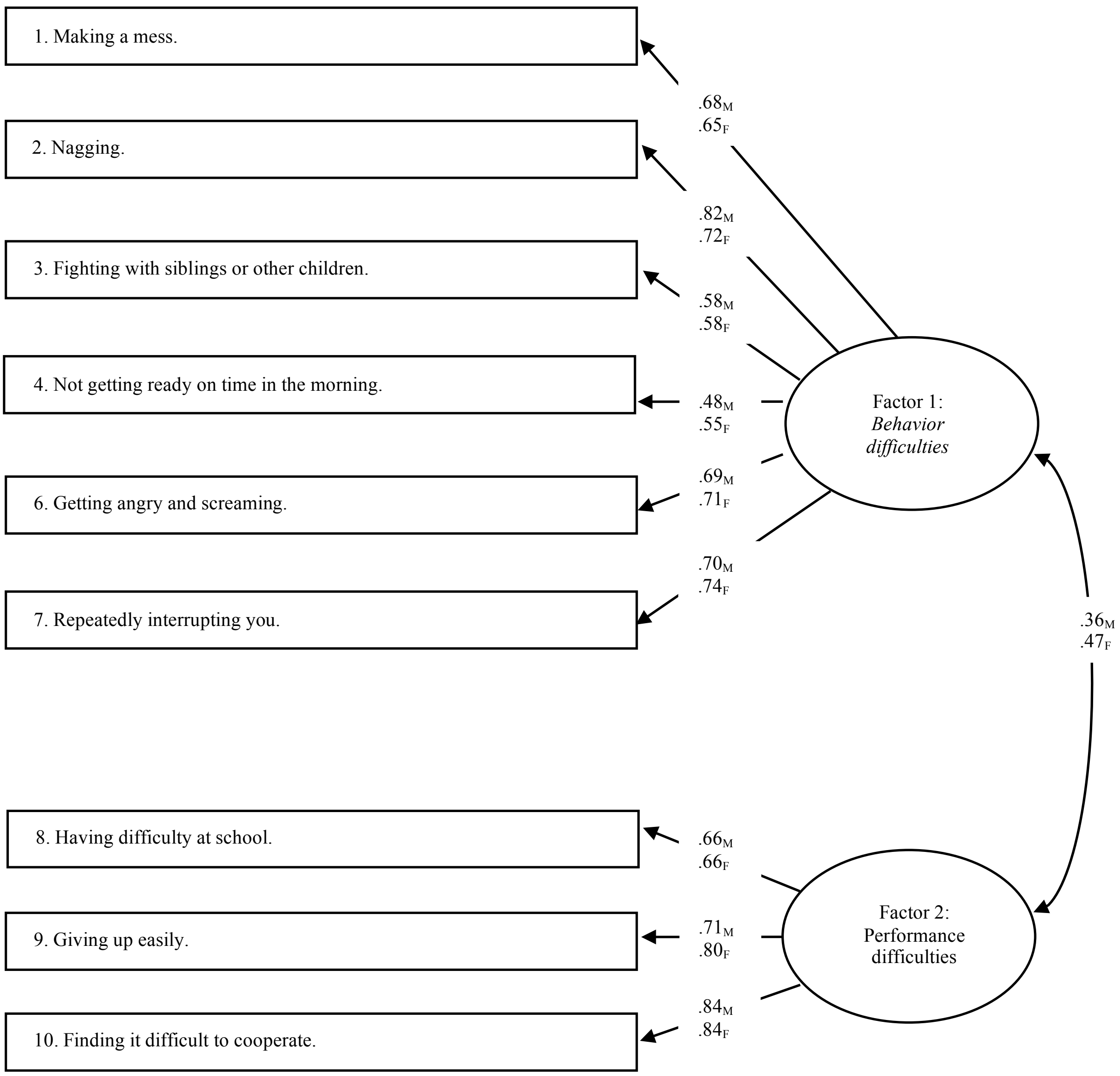

Fig. A4

Factor structure of the perceived susceptibility scale for mothers and fathers. Standardized factor loadings. Model fit: WLSMV χ^2 (74) = 126.05, $p < .001$; CFI = .980; RMSEA = .049 95%CI (.034-.064); all models based on N = 284 fathers and N = 286 mothers; all factor loadings significant at $p < .001$; correlation between latent constructs significant at $p < .001$

How bad do you think it would be if your child were to engage in (/experience) each of the following behaviors (/difficulties) two years from now?

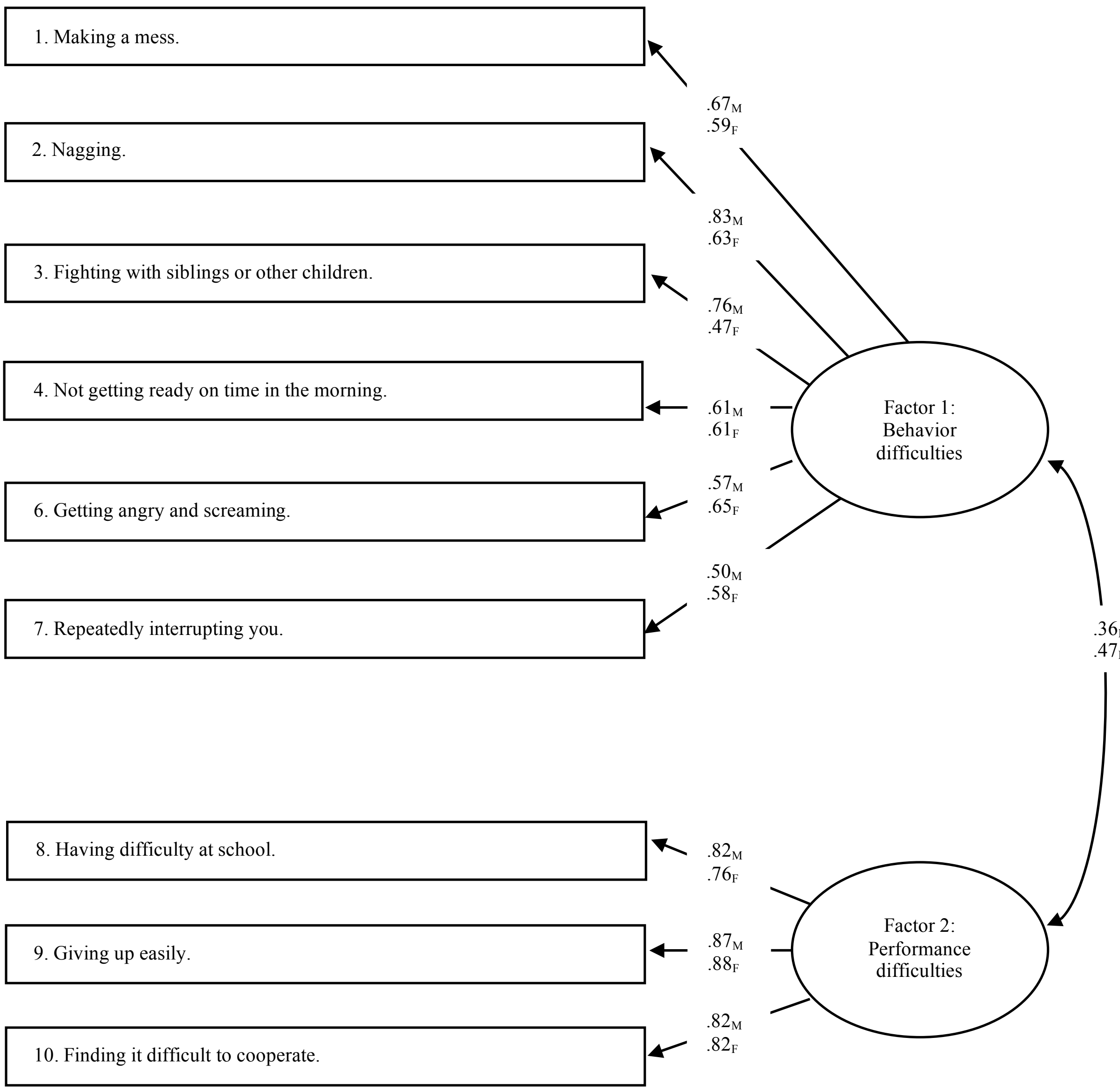

Fig. A5

Factor structure of the perceived severity scale for mothers and fathers. Standardized factor loadings. Model fit: WLSMV χ^2 (71) = 130.37, p <.001; CFI = .978; RMSEA = .054 95%CI (.039 – .068); all models based on N = 284 fathers and N = 286 mothers; all factor loadings significant at p < .001; correlation between latent constructs significant at p < .001

How much do you agree with each of the following statements?

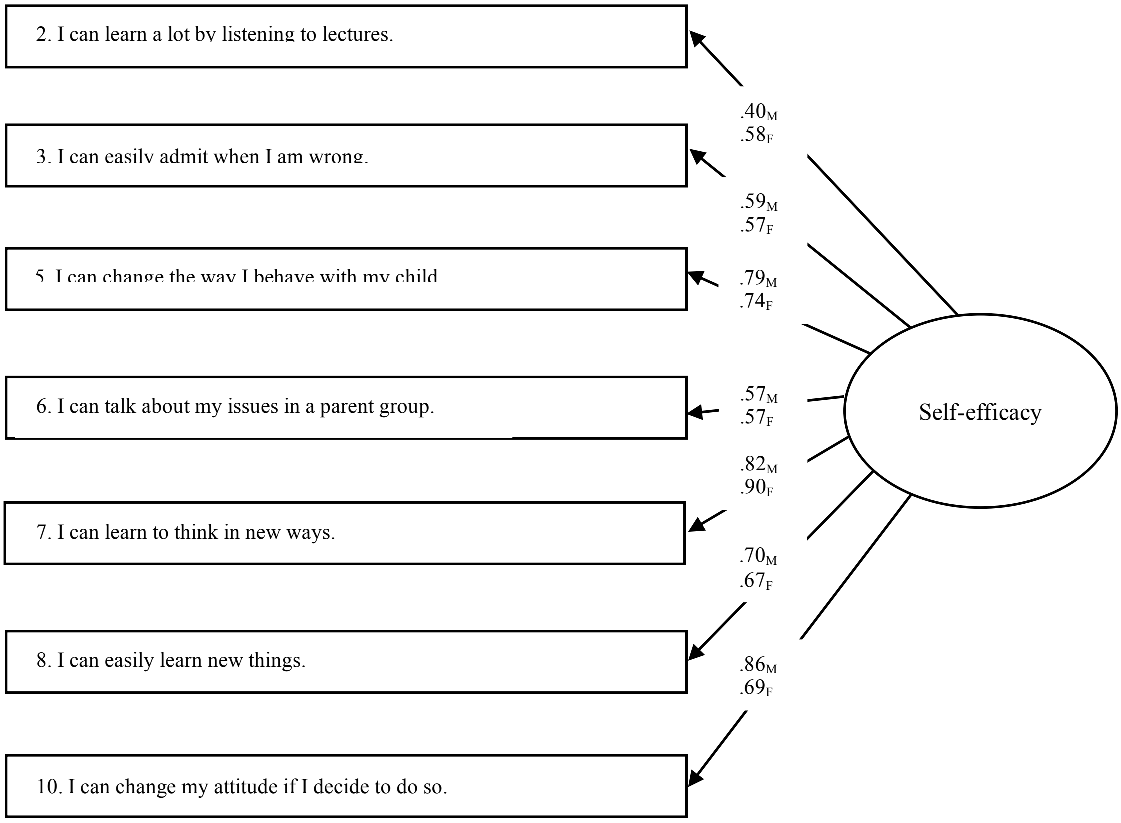

Fig. A6

Factor structure of the perceived self-efficacy scale for mothers and fathers. Standardized factor loadings. Model fit: WLSMV χ^2 (39) = 147.79, p < .001; CFI = .948; RMSEA = .08 95%CI (.082 – .115); all models based on N = 284 fathers and N = 286 mothers; all factor loadings significant at p < .001
